# Idiopathic dilated cardiomyopathy: possible triggers and treatment strategies

**DOI:** 10.1007/s12471-012-0285-7

**Published:** 2012-05-22

**Authors:** M. Hazebroek, R. Dennert, S. Heymans

**Affiliations:** 1 Department of Cardiology, CARIM, University Hospital Maastricht, Maastricht, the Netherlands; 2 Interuniversity Cardiology Institute of the Netherlands, Utrecht, the Netherlands; 3 Center for Heart Failure Research Cardiovascular Research Institute Maastricht (CARIM), University Hospital Maastricht, P. Debyelaan 25, 6229 HX Maastricht, the Netherlands

**Keywords:** Idiopathic dilated cardiomyopathy, Possible triggers, Viral-mediated, Inflammatory mediated, Treatment strategies

## Abstract

Despite recent advances in the management of patients with heart failure, morbidity and mortality rates remain high. Common causes of heart failure are ischaemic heart disease, uncontrolled hypertension and valvular disease. However, in up to 50 % of the cases its exact cause remains initially unknown; this condition is called idiopathic dilated cardiomyopathy (DCM). Improved diagnostic methods, most notably the advancements in molecular and immunohistological biopsy techniques and genetic research, have endorsed a new era in the diagnosis and classification of patients with idiopathic DCM. These insights have led to novel aetiology-based treatment strategies and improved outcome. The present article will briefly discuss all causes of DCM with a special focus on inflammatory- and virus-mediated forms of DCM.

## Introduction

Despite recent advances in the management of patients with heart failure, morbidity and mortality rates remain high. It is the only cardiovascular disease that has increased in prevalence over the last 20 years [1]. With over 200,000 patients suffering from heart failure in the Netherlands, it continues to impose a great burden on the health care system. Common causes of heart failure are ischaemic heart disease, uncontrolled hypertension and valvular disease. However, in up to 50 % of the cases its exact cause remains initially unknown; this condition is called idiopathic dilated cardiomyopathy (DCM) [2]. Patients with idiopathic DCM are relatively young, ranging between 20 and 60 years, and often still in the prime of their lives. To discriminate different aetiologies of DCM, specific diagnostic methods including serum markers, cardiac biopsies and genetic screening are crucial to improve care and outcome in these patients.

## Triggers of idiopathic dilated cardiomyopathy

**Fig. 1:**
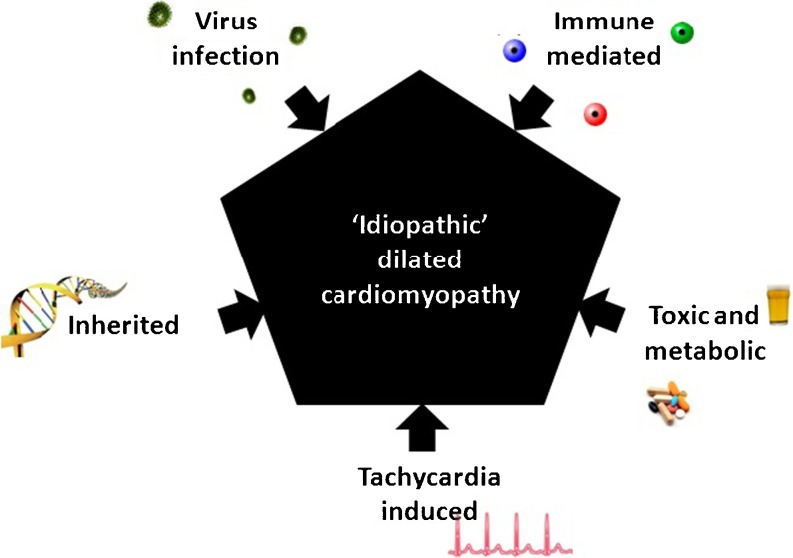
Possible triggers of idiopathic dilated cardiomyopathy

DCM may be the consequence of a wide variety of causes, including virus-mediated disease, immune dysregulation, toxic and metabolic, inherited, and tachycardia-induced conditions (Fig. 1) [2, 3]. We will briefly discuss all the possible triggers of DCM with a special focus on inflammatory- and virus-mediated forms of DCM.

### Toxic and metabolic triggers

Toxic cardiomyopathy results from toxic exposure of a variety of cardiotoxic agents, most notably alcohol and chemotherapeutic drugs. Metabolic cardiomyopathies can be caused by a wide spectrum of pathological metabolic conditions (Table 1) [4].

**Table 1:** Classification of toxic and metabolic cardiomyopathies

Infiltrative	Amyloidosis (primary, familial autosomal dominant, senile, secondary forms) Gaucher disease, Hurler’s disease and Hunter’s disease
Storage	Haemochromatosis, Anderson-Fabry disease, glycogen storage disease (Pompe), Niemann-Pick disease
Nutritional deficiencies	Beriberi (thiamine), pellagra, scurvy, selenium, carnitine, nutritional disorder such as kwasiorkor
Endocrine	Diabetes mellitus, hyperthyroidism, hypothyriodism, hyperparathyroidism, pheochromocytoma
Toxicity	Alcohol, drugs (cocaine, catecholamines, lithium, phenothiazines, methysergide), heavy metals (cobalt, lead, arsenic) and chemical agents
Consequence of cancer therapy	Anthracyclines (doxorubicin, daunorubicin), cyclophosphamide and radiation

### Tachycardia-induced cardiomyopathy

Tachycardia-induced cardiomyopathy (TIC) is caused by sustained rapid ventricular rates and is one of the well-known forms of reversible myocardial diseases after the normalisation of heart rate. It may follow any type of chronic cardiac arrhythmia: supraventricular tachyarrhythmias, ventricular tachycardia, and frequent premature ventricular complexes. The diagnosis of TIC remains difficult since cardiomyopathy and tachycardia are often identified simultaneously [5]. In up to 70 % of the cases, a tachycardia is the symptom and not the cause of an underlying cardiomyopathy.

### Inherited triggers

A gene mutation may be the cause of DCM in up to 30 % of the cases [3], therefore cardiogenetic screening is crucial in idiopathic DCM patients. The most common mode of inheritance is autosomal dominant transmission, although less common forms including X-linked, autosomal recessive and mitochondrial inheritance have also been described. So far, 40 genes have been described underlying inherited forms of idiopathic DCM. The mutated genes predominantly encode two major subgroups: cytoskeletal and sarcomeric proteins. The cytoskeletal proteins include dystrophin, desmin, lamin A/C, δ-sarcoglycan, β-sarcoglycan and metavinculin. In case of the sarcomeric proteins they include β-myosin heavy chain, myosin-binding protein C, actin, α-tropomyosin, and cardiac troponin T and C. Furthermore, phospholamban, tafazzin and the sodium-channel gene *SCN5A* have also been reported [3].

With the upcoming awareness of the importance of a patient’s genetic background and the increasing numbers of mutated genes found, genetic testing for idiopathic DCM is becoming more common in clinical practice. In turn, this is an opportunity to unravel the molecular complexity of inherited DCM and develop possible disease-modifying therapies in the future.

### Virus infection

Virus infection may cause acute and chronic myocarditis, and viral persistence has been linked to the development of ‘idiopathic’ DCM. Until the 1990s, the most frequently reported viruses in patients in the developed countries were adenoviruses and enteroviruses. Recently, parvovirus B19 (B19V) and human herpes virus-6 are increasingly found in a significant percentage of patients diagnosed with both acute and chronic cardiomyopathy. While up to 50 % of young adults and up to 90 % of the elderly have been infected with these cardiotropic viruses, only an ‘unlucky few’ develop cardiac sequelae [6].

Endomyocardial biopsies (EMB) are the golden standard for the diagnosis of virus presence and inflammation in the heart. In the acute phase the living virus actively replicates within the myocardium, causing damage to cardiomyocytes and endothelial cells, in turn triggering the innate immune response. In most patients, this leads to viral clearance with subsequent adequate downregulation of the immune response resulting in a healthy recovered heart. However, in some patients the immune response is insufficient and clearance of the virus is not achieved. This may lead to viral persistence, causing progressive myocyte damage which may ultimately progress to biventricular dilatation with cardiac failure [6]. Therefore, a certain genetic background appears to be a prerequisite to developing clinical symptoms of myocarditis and/or progression to virus-induced DCM. This is illustrated by viral proteases which may cleave dystrophin, a cytosolic protein which is also affected in patients with Duchenne muscular dystrophy, leading to progression of heart failure symptoms in these patients.

### Immunological triggers

The role of viral infections in autoimmune disease has been a topic of interest for over a century. There are two general mechanisms by which viruses may induce autoimmunity. Firstly, by providing or presenting the disease-initiating antigen inducing the innate immune response, and secondly by direct myocardial involvement of immune-mediated inflammatory damage.

While viral clearance by the innate immune response may improve clinical outcome, detrimental secondary effects may be triggered after the primary infection. Primed T-cells detect viral antigens and destroy infected cardiomyocytes through Fas/Fas ligand, TNF-alpha, cytokine and perforin pathways. In addition, some host myocardial cellular antigens may share epitopic similarities (molecular mimicry) with viral antigens, and may therefore induce an autoimmune response that can sustain the inflammatory response even after initial viral elimination. This autoimmune response induces a chronic inflammatory phase leading to immune-mediated myocyte damage.

Secondly, besides a primary virus trigger for immune dysregulation, also organ-specific and systemic immune-mediated diseases such as Wegener granulomatosis, Churg-Strauss syndrome or sarcoidosis are known to directly affect the heart. Cardiac involvement is one of the complications that substantially contribute to mortality and morbidity in patients with systemic inflammatory diseases. In addition, increased serum markers for immune activation and autoantibodies (i.e. α/β-myosin heavy chain, myosin light chain, troponin) may be detected in patients with autoimmune-mediated inflammatory disease [7]. General screening for this immune dysregulation with subsequent increased serum markers is performed by measuring atrial natriuretic factor, soluble interleukin-2 and neopterin levels.

## Treatment strategies for idiopathic DCM

The goals of treatment in patients with idiopathic DCM are to improve survival, slow disease progression, minimise risk factors, and alleviate symptoms. A standard heart failure regimen with lifestyle modifications should be initiated in all patients with this cardiac disease, including ACE inhibitors, angiotensin-II receptor antagonists, beta-blockers, diuretics, aldosterone antagonists, and digitalis. In some select patients known with rhythm disturbances and/or increased risk of sudden cardiac death, resynchronisation therapy combined with an implantable cardioverter device should be considered to reduce morbidity and mortality.

Besides standard heart failure therapy, determination of the aetiology of idiopathic DCM is essential to initiate treatment strategies if possible. In some cases, the specific condition can be addressed, such as alcohol abuse and chemotherapy, to prevent disease progression. Catheter ablation should be considered in idiopathic DCM patients with frequent monomorphic PVCs (>10 % of QRS complexes), sustained rapid supraventricular tachyarrhythmias or ventricular tachycardias.

In case of a previously unknown inflammatory heart disease, distinction between virus-positive and virus-negative inflammatory DCM, performed with EMB, is crucial. Currently, sparse studies have investigated the role of antiviral therapy in an inflammatory DCM with viral presence. Recently, a pilot study with high-dose intravenous immunoglobulin (2 g/kg), known to especially eliminate the most frequently found B19V in cardiac biopsies, has demonstrated favourable effects on both cardiac function as well as virus elimination in virus-positive inflammatory DCM patients [8]. Therefore, a randomised, double-blind, placebo-controlled study has been initiated in patients with unexplained heart failure related to a significant B19V myocardial persistence (>200 copies/μg DNA).

In the case of virus-negative inflammatory DCM patients, a randomised controlled trial has recently demonstrated the beneficial effects of immunosuppressive therapy on myocardial function [9]. Patients received either prednisone and aziathioprine for 6 months (43 patients) or placebo (42 patients) in addition to conventional heart failure therapy for 6 months. Although other studies have also investigated the effects of immunosuppression in autoimmune-mediated inflammatory DCM (diagnosed by human leucocyte antigens or circulation auto-antibodies), only Frustaci et al. confirmed the absence of cardiotropic viruses in endomyocardial biopsies within his study population. Additionally, immunoadsorption therapy with subsequent immunoglobulin substitution has proven to be an effective immunomodulatory regimen in inflammatory DCM patients, although the presence of myocardial virus genome was also not addressed in these trials [10].

In conclusion, a detailed diagnosis of virus presence, inflammation, autoimmune dysregulation and genetic background is needed in idiopathic DCM patients to develop better classification methods, more aetiology-based treatment strategies and sophisticated prognostic models in the near future.
